# Depression history and memory bias for specific daily emotions

**DOI:** 10.1371/journal.pone.0203574

**Published:** 2018-09-07

**Authors:** Emily J. Urban, Susan T. Charles, Linda J. Levine, David M. Almeida

**Affiliations:** 1 Department of Psychological Science, University of California Irvine, Irvine, California, United States of America; 2 Department of Human Development and Family Studies, The Pennsylvania State University, University Park, Pennsylvania, United States of America; University of Pittsburgh, UNITED STATES

## Abstract

Reports of emotions experienced over the past week can be influenced by memory bias, which is more pronounced for people with depression. No studies, however, have examined memory bias for specific emotion clusters (e.g., sadness, anxiety, and anger) experienced on a day-to-day basis among people with depression or a history of depression. Participants (*N* = 1,657) from the Midlife in the United States Study were assessed for depression. Approximately 6 months later, participants reported their emotional experiences for 8 days and recalled these experiences on the final day. Differences in recalled and reported emotion were compared between participants with and without a history of depression. Participants overestimated experience only of negative emotions, particularly anger, and this negativity bias was greatest for participants with a history of depression. Feelings related to anger were prone to greater overestimation than sadness or anxiety. These findings emphasize the role of memory bias in retrospective reports of specific emotions and illustrate the presence of an amplified memory bias among people who are at a greater risk for recurrent depressive episodes.

## Introduction

Retrospective self-reports of emotional experiences are subject to errors and bias. People often overestimate the extent to which they experienced past emotions, a phenomenon referred to as the memory-experience gap [1]. The size of this gap depends upon characteristics of the individual (e.g., [2]) and the valence of emotion recalled. For example, the occurrence of both positive and negative emotion tends to be overestimated retrospectively [3], but overestimation of negative emotion is typically more pronounced (e.g., [1, 3]). This pattern has been interpreted in terms of an adaptive evolutionary advantage, as negative events pose a greater threat to survival compared to positive events [1, 4]. Overestimating how negative an event was in memory motivates a person to avoid similar situations in the future even more so than remembering the negative event accurately. Biases in memory for emotion also result from changes in people’s cognitive appraisals of events over time. Memory for emotion becomes less accessible over time. When people attempt to remember how they felt in the past, they often draw on their current cognitive appraisals of emotion-eliciting events to help them reconstruct how they must have felt [5]. The more people’s cognitive appraisals have changed over time, the greater the difference between remembered and experienced emotion [6].

### Memory bias and depression

The memory-experience gap may also vary based on depression status, as depression is often associated with cognitive biases [7–8]. Although people tend to retrieve a greater number of positive than negative autobiographical memories regardless of depression status, people in negative or depressed moods tend to exhibit a negativity bias, attending to and retrieving disproportionately more negative than positive memories relative to those who are feeling more positive or who are not depressed [9–12]. Diagnoses of depression are often based on a person’s recollections of his or her recent emotional experiences and therapeutic treatment of depression typically involves a patient’s report of how they felt over the last week. Because of the biases in cognitive processing exhibited among people with depression, however, retrospective reports might not provide an accurate representation of the patient’s daily emotional experiences.

People who have had a depressive episode in the past are often characterized by stable, trait-like qualities that predispose them to experience additional depressive episodes in the future. According to one large multinational study, 75% of people who have a depressive episode will have at least one more episode in their lifetimes [13]. One of the strongest predictors of depressive episode recurrence is the presence of subclinical depressive symptoms after recovering from the last depressive episode [14]. This means that even after a person has recovered from a depressive episode, they are likely to exhibit similar symptoms, such as depressed mood and cognitive deficits, albeit to a lesser magnitude. The continued experience of depressive symptoms after recovering from an episode, including a negativity bias in memory for previously experienced emotions, could play a role in the high recurrence rate of depression. Therefore, assessing daily emotions and negativity bias for past emotions among people who have recently experienced a depressive episode is extremely important in understanding how previous depressive episodes make one more susceptible for future episodes.

By comparing daily emotions with retrospective reports of the same emotions, we can examine the extent to which memories of elevated negative emotion in those with recent depression reflect actual, everyday experience rather than the memory-experience gap or the presence of other cognitive biases. This would allow for a more complete picture of everyday symptoms among those with recent depression and thus more informed clinical interventions. The current study examines specific clusters of emotions (e.g., sadness, anxiety, and anger) people experience in daily life, their recollections of these emotional experiences, and how these reports vary across people with and without a recent history of depression.

To our knowledge, no studies have compared the memory-experience gap in emotions experienced in daily life between people with and without a recent history of depression. Only two studies to our knowledge have compared the memory-experience gap in emotions experienced in daily life between people with and without current depression, one including 51 participants with and without a depression diagnosis [15] and the other 120 participants who varied in symptoms of anxiety and depression [16]. Both studies, which examined differences in composite measures of positive and negative emotion, found that people with depression experience more negative and less positive emotion in daily life than controls. Furthermore, even though participants in both studies tended to overestimate emotions (both positive and negative), people with depression tended to overestimate negative emotions to a greater extent and overestimate positive emotions to a lesser extent [16] or similar extent [15] than people without depression. The current study expands on this research to examine the experience and memory of specific clusters of emotions across people with and without a recent history of depression.

### Specific emotions experienced within depression

Clinical inventories and diagnostic criteria assessing depression focus on a person’s *general mood* (i.e., significant levels of depressed mood or anhedonia) over a span of weeks, not on specific types of emotions [17]. The few studies that examine daily emotions of people with depression similarly tend to focus on composite measures of positive and negative emotions (e.g., [18]). Measuring the prevalence of specific clusters of emotions (e.g., sadness, anxiety, and anger), and how these emotions are remembered, could aid in understanding the daily experience of people who are prone to depression.

Feelings of sadness, loneliness, and feeling “blue” over a span of weeks are considered hallmark symptoms of depression [19–20], and therefore would be expected when measured on the daily level. Feelings of anxiety are also frequent among people with depression, but are not included in the diagnostic criteria. Anxiety and depressive disorders share some common symptoms, such as insomnia, psychomotor agitation, and difficulty concentrating; additionally, they commonly co-occur [21–22]. Thus, extensive research findings support the view that elevated sadness and anxiety, and related symptoms, are common for individuals with a history of depression and likely play a prominent role in their daily lives.

Anger is increasingly thought to also accompany the experience of depression. However, clinical diagnoses only recognize anger as a hallmark symptom of depression in children and adolescents [17], and research documenting anger among adults with depression has only recently begun to grow. A handful of studies over the years have documented the presence of anger and irritability among people with depression [23–26]. One research group found that anger, along with sadness and anxiety, accounted for a significant portion of the variance in a depressive diagnosis among three independent samples [27–28]. Moreover, symptoms of irritability were clinically relevant in between 20 to 27% of participants with a diagnosis of depression.

Based on the research reviewed in this section, sadness, anxiety, and anger are likely to play a large role in the daily lives of people with a recent history of depression. Examining how people with and without a history of depression remember these emotions as they occur in everyday life would elucidate the relationship between depression and accuracy in retrospective reports of emotion and shed light on how people with a history of depression might be at risk for a future depressive episode.

### The current study

The current study builds on prior findings by using a large national daily diary study to examine how daily emotional experiences, and specifically different clusters of emotions, compare to recollections of the same emotional experiences during that week, and how this varies based on history of depression.

Based on prior research, we predicted that those with a recent history of depression would report diminished positive and elevated negative emotion during the diary study compared to those with no depression history. Regarding experiences of sadness, anxiety, and anger, we predicted that all negative emotions would be elevated among participants with a recent history of depression relative to those without the diagnosis, but that these differences would be most pronounced for sadness. The prediction that anger would be higher among the group with a recent history of depression was based on a small but growing literature on the experience of anger and irritability among adults with depression [23–26].

Regarding memory bias, we further hypothesized that participants would overestimate the frequency of emotional experiences, and particularly negative emotions, based on past findings on memory for intensity and frequency of emotion (e.g., [1, 3]). Given the association between depression and biases in cognitive processing, we expected people with a history of depression to overestimate their experiences of negative emotions to a greater extent (particularly experiences of sadness given its prevalence in depression), and to overestimate experiences of positive emotions to a lesser extent, than those without a recent history of depression.

## Method

### Participants

Participants included people from the Midlife in the United States Study II (MIDUS II) who also participated in the National Study of Daily Experiences (NSDE II; *N* = 2022). MIDUS II took place between 2004 and 2006. People were excluded from the primary analyses if they were missing information for a history of depression diagnosis (*n* = 180), were missing data on their education level (*n* = 3), or if they did not complete the end-of-week emotion recall portion of the study at the conclusion of data collection (*n* = 182). The remaining 1,657 participants who composed the present sample ranged in age from 33–84 (*M* = 56.67) and 56.2% were female. More than two-thirds of the participants had more than a high school education (70.8%) and most were Caucasian (92.5%, 2.9% African American, 1.4% Native American or Alaska Native Aleutian Islander/Eskimo, 0.5% Asian, 2.5% other ethnicities, 0.2% did not know, and 0.1% did not specify).

Given that MIDUS included information about depression history at two time points approximately 10 years apart (MIDUS I was collected between 1995–1996 and MIDUS II was collected between 2004–2006), we explored separately whether people with chronic depression, as calculated by symptoms that placed them in the depression category at both waves, would report the highest levels of negative emotions and even greater discrepancy between the emotion reporting types compared to people who were newly depressed (i.e., only meeting criteria at the second wave). The results of these analyses can be found in the supporting information section.

### Research design

All participants first completed the MIDUS II questionnaires which assessed demographic information and history of depression in addition to other social and health related characteristics. An average of six months later, participants began the NSDE II by reporting their experiences over the past 24 hours (typically at the end of the day) across eight consecutive days. End-of-day reports of emotional experiences have been found to correlate highly with momentary reports, demonstrating the validity of using end-of-day reports to gather accurate information on daily emotional experiences [29–30]. On the final day of the diary study, participants provided estimates of their average emotional experiences across the past seven days. Statistical analyses were conducted using SPSS version 20 (IBM Corp., released 2011, Armonk, NY).

### Measures

#### Demographic information

Demographic information collected in the MIDUS II questionnaire included age, gender, and highest level of education completed. Education was rated on a scale from 1 *(no school/some grade school)* to 12 *(professional degree)*.

#### Recent history of depression

During MIDUS II, telephone interviewers asked participants questions that screened for a depression disorder using the Composite International Diagnostic Interview Short Form (CIDI-SF) [31]. This measure was developed based on the DSM-III-R criteria [32] and used by the World Health Organization. Participants who reported feeling sad, blue, or depressed almost every day for at least most of the day for two weeks or more in a row during the past 12 months and who also experienced at least four additional depressed mood or anhedonia symptoms (e.g., trouble concentrating, loss of energy, loss of interest) during this time were coded as having met the criteria for a depressive disorder. Because this measure was assessed prior to the daily diary study and asked about the past 12 months, we could not ascertain whether people were currently depressed or were no longer depressed during the daily diary study. Therefore, we refer to this measure as indicating a recent history of depression. Of the 1657 participants in the current study, 158 (9.5%) met the criteria for having a history of depression.

#### Daily experienced positive and negative emotion

Each day for eight days, participants in NSDE II reported how much of the time during that day they had felt each of 13 positive and 14 negative emotions, using a scale that ranges from 0 *(none of the time)* to 4 *(all of the time*). These items were selected from the Positive and Negative Affect Schedule [33] and the Non Specific Psychological Distress Scale [34].

Positive emotion items included feeling cheerful, in good spirits, extremely happy, calm and peaceful, satisfied, full of life, enthusiastic, attentive, proud, active, close to others, like you belong, and confident (α = .96). For each individual, responses for each question were averaged together for each day. Next, these eight daily values were averaged to produce one value reflecting the average frequency of positive emotions experienced during the week. We also ran a principal component analysis (PCA) on all participants who completed NSDE II (*n* = 2022) using an oblique rotation on the 13 positive emotion items to examine whether these emotions separated into different factors. Results revealed one underlying factor representing general positive emotion (Eigenvalue = 9.35; the second factor Eigenvalue = 0.72), which accounted for 71.93% of the variance in positive emotion ratings. Thus, analyses were limited to one measure of positive emotion.

Negative emotion items included feeling worthless, so sad nothing could cheer you up, nervous, restless or fidgety, hopeless, that everything was an effort, afraid, jittery, irritable, ashamed, upset, lonely, angry, and frustrated, (α = .90). As done for positive emotions, responses to these 14 negative items were averaged together for each day. These eight daily scores were then averaged to create one score reflecting the average frequency of negative emotions experienced during the week. A PCA of the 14 negative emotion items from NSDE II participants using an oblique rotation revealed three components which together accounted for 65.1% of the variance (see Table 1 for factor items and factor loadings). The first component (Eigenvalue = 6.48) accounted for 46.29% of the variance in negative emotion and consisted of the items related to sadness. The second component (Eigenvalue = 1.60) accounted for 11.41% of the variance in negative emotion and included items related to anger. The third component (Eigenvalue = 1.04), which accounted for 7.41% of the variance, consistent of items related to anxiety. Emotions within the sad, anger, and anxiety factors were averaged within each day, and then were averaged across the week resulting in three separate scores for sadness, anger, and anxiety emotions experienced during the week.

**Table 1:** Pattern matrix of negative affect items.

Negative Emotion Variable	Sadness	Anger	Anxiety
Hopeless	**.93**	-.04	-.14
So sad nothing could cheer you up	**.89**	.01	-.05
Worthless	**.79**	.05	.02
Lonely	**.60**	.02	.26
Ashamed	**.56**	-.06	.05
Everything is an effort	**.50**	-.16	.06
Angry	.01	**-.91**	-.10
Frustrated	.05	**-.84**	.04
Upset	.10	**-.81**	.03
Irritable	-.08	**-.79**	.18
Jittery	-.04	.03	**.89**
Nervous	.01	-.09	**.82**
Restless/Fidgety	.03	-.19	**.67**
Afraid	.35	.03	**.48**

*Note*. Sadness, Anger, and Anxiety factors accounted for 46.29%, 11.41%, and 7.41% of variance in Negative Affect ratings, respectively. A principal component analysis with an oblique rotation was used.

#### Recalled emotion

On the eighth diary day, participants were asked to think over the past week and rate how much of the time they felt each of the same 13 positive and 14 negative emotion items on a scale from 0 *(none of the time)* to 4 *(all of the time*). This measure of recalled emotion has been used in previous work examining age differences in temporal reports of emotional well-being (see [35]). The 13 positive emotion frequencies were averaged, resulting in one recalled positive emotion score (α = .95). Similarly, the 14 negative emotion frequencies were averaged to get one recalled negative emotion score (α = .88).

As with the average of daily emotions, two separate PCAs were conducted to examine the factor loadings of positive and negative emotions recalled from the week. Results were similar to those of the daily experienced emotions. Positive emotions loaded onto one component only (Eigenvalue = 8.27; second factor Eigenvalue = 0.75) which explained 63.63% of the variance in positive emotion reports. Three components found in the factor analysis of recalled negative emotions consisted of the same emotion items found in the analysis of daily emotions, although fear loaded almost equally on both the sadness and anxiety components (.38 and .35 respectively). For the purposes of the current research, the fear item was included only in the anxiety component to match the experienced emotion groupings. Together, recalled sadness (Eigenvalue = 5.57; variance accounted for 39.78%), anger (Eigenvalue = 1.72; variance accounted for 12.31%), and anxiety (Eigenvalue = 1.05; variance accounted for 7.46%) accounted for 59.55% of the overall variance in recalled negative emotion. Recalled frequencies of each specific emotion cluster (sadness, anger, and anxiety) were calculated by averaging the recalled emotions within each cluster.

## Results

The prevalence rate for people meeting criteria for depression in MIDUS II (*n* = 158, 9.5%) is similar to other 12-month prevalence estimates of depression in the United States (e.g., 10%) [13]. Comparing those who met criteria to those who did not (*n* = 1499), participants who had a recent history of depression were younger, *t*(201.05) = 5.49, *p* < .001, *r* = .36, 95% *CI* [.32, .40], and more likely to be female, *χ*^2^(1) = 15.33, *p* < .001, *φ* = .096, but did not vary in levels of education, *t*(1655) = 1.53, *p* = .127, *r* = .04, 95% *CI* [-0.01, 0.09]. Given these associations, age and gender were included as covariates in all analyses. Education was also included given that people at greater risk for depression are more likely to have lower levels of education [36], although this was not the case in our sample. All analyses were also conducted without age, gender, and education as covariates. Excluding these covariates did not change the nature of the results.

### Experience and memory of positive and negative emotion

Our first model (full results listed in Table 2) tested the hypotheses regarding differences in positive and negative daily experiences by depression group, and whether the difference between average daily reports and recollection of emotion (i.e., memory bias) was more or less pronounced for people with a history of depression. A repeated measures general linear model (RM GLM) included the within-person differences of emotion (positive vs. negative) and report type (daily average vs. end-of-week recollection) by the between-subject factor of depression history (with and without) with age, gender, and education as covariates. We also explored potential interactions with the covariates. We followed up any significant interactions with post-hoc multiple comparisons, t-tests, and correlational analyses. All confidence intervals are reported at the 95% level.

**Table 2:** Effects of history of depression on experience and recall of positive & negative affect.

		*F*(1, 1652)	*Partial η*^*2*^ [90% *CI*]
Within Subjects Main Effects	Valence	166.47***	.092 [.071, .114]
	Report Type	91.44***	.052 [.036, .071]
Between Subjects Main Effects	Age	18.44***	.011 [.004, .021]
	Gender	4.96*	.003 [.002, .009]
	Education	3.37	.002 [.000, .007]
	HoD	19.72***	.012 [.005, .022]
Two-way Interactions	Valence*Report Type	18.14***	.011 [.004, .021]
w/ Valence	Age*Valence	59.63***	.035 [.021, .051]
	Gender*Valence	0.38	.000 [.000, .003]
	Education*Valence	6.83**	.004 [.000, .011]
	HoD *Valence	123.40***	.070 [.051, .090]
w/ Report Type	Age*Report Type	53.12***	.031 [.019, .046]
	Gender*Report Type	2.26	.001 [.000, .006]
	Education*Report Type	5.37*	.003 [.000, .009]
	HoD *Report Type	26.01***	.015 [.007, .027]
Three-way Interactions with Valence*Report Type	Age*Valence*Report Type	7.03**	.004 [.001, .011]
	Gender*Valence*Report Type	12.21***	.007 [.002, .016]
	Education*Valence*Report Type	5.36*	.003 [.000, .009]
	HoD*Valence*Report Type	13.85***	.008 [.003, .017]

*Note*. Valence refers to positive compared to negative affect. Type refers to daily emotion reports (averaged across the week) compared to recalled emotion (made at the end of the week). HoD = History of Depression. *CI* = Confidence interval.

***p < .001.

**p < .01.

*p < .05.

**Fig 1:**
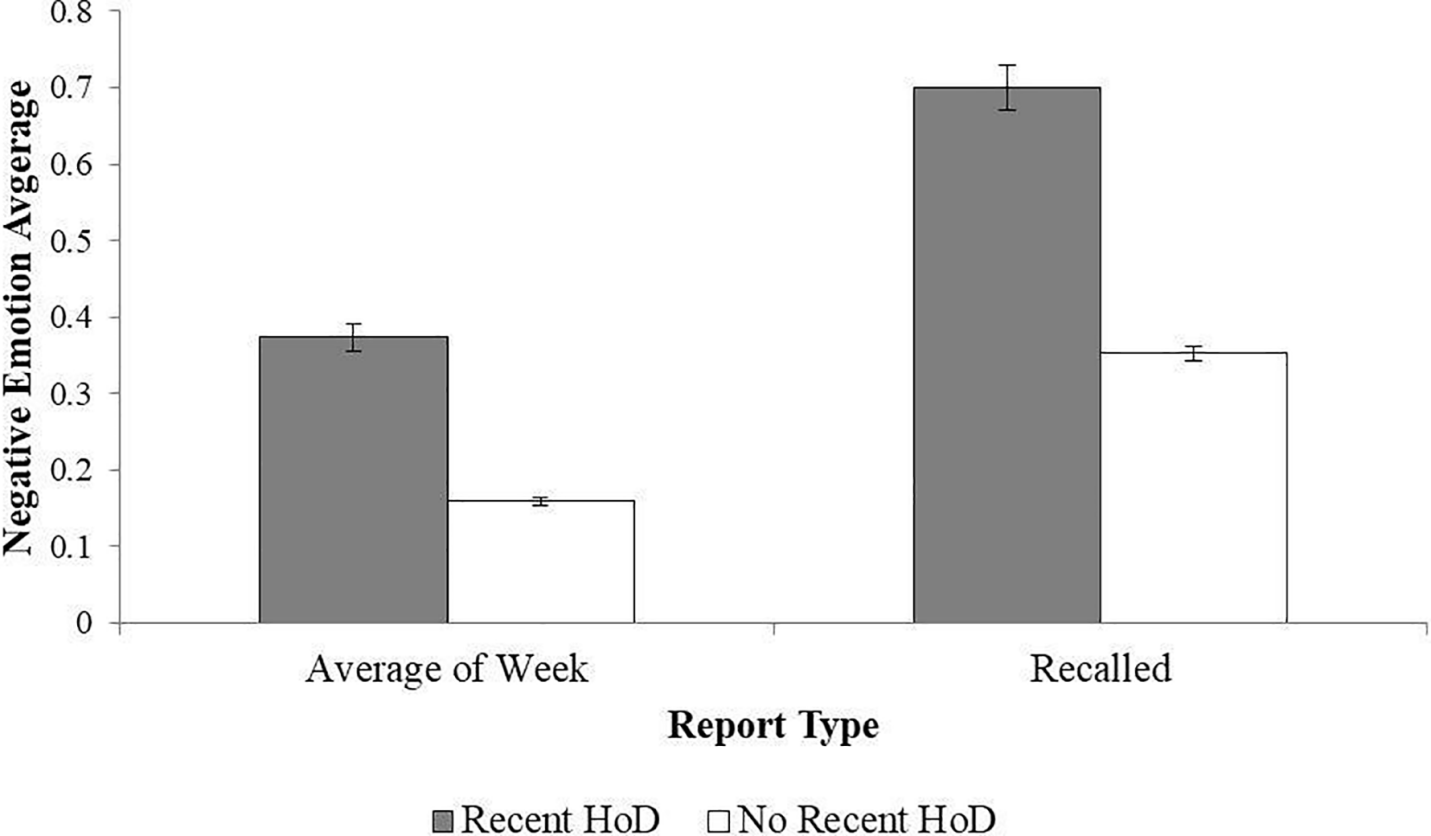
Negative emotion by recent history of depression. *Note*. Error bars represent standard error values. HoD = History of Depression. The model is adjusted for gender and evaluated at the following values: Age = 56.67, Education = 7.39.

A significant main effect of depression group was qualified by a three-way interaction between valence, report type, and depression history group, (see Fig 1 for a graph of negative emotion). Both groups reported more positive than negative emotion during the week. Participants with a history of depression experienced lower levels of daily positive emotion and higher levels of daily negative emotion compared to those without a history of depression. When comparing recollections of emotions to daily reports, we found that recollections of positive emotion were not significantly greater than daily reports for either group. Both groups overestimated the frequency with which negative emotion was experienced during the week, indicated by significantly higher recalled negative emotions than daily emotions, but this bias was significantly greater for participants with a history of depression.

Taken together, results confirm predictions that people with a history of depression experience more negative and less positive emotion in daily life, and overestimate the frequency of negative emotion in their recollections to a greater degree compared to people without a history of depression. Although people with a history of depression also experienced lower levels of positive emotion than those without, positive emotions were not significantly over- or under- estimated by either group.

With respect to covariates, older age was associated with experiencing more positive emotion, *r* = .20, *p* < .001, *CI* [.15, .25], less negative emotion, *r* = -.16, *p* < .001, *CI* [-.21, -.11], and less memory bias for negative emotion, *r* = -.21, *p* < .001, *CI* [-.26, -.16]. Furthermore, women tended to overestimate negative emotion significantly more than men, *t*(1655) = -4.15, *p* < .001, *r* = .10, *CI* [.05, .15], as indicated by a significant interaction between valence, report type and gender. Finally, having more education was related to experiencing less frequent positive emotion, *r* = -.07, *p* = .003, *CI* [-.12, -.02].

#### Experience and memory bias of specific negative emotions

**Fig 2:**
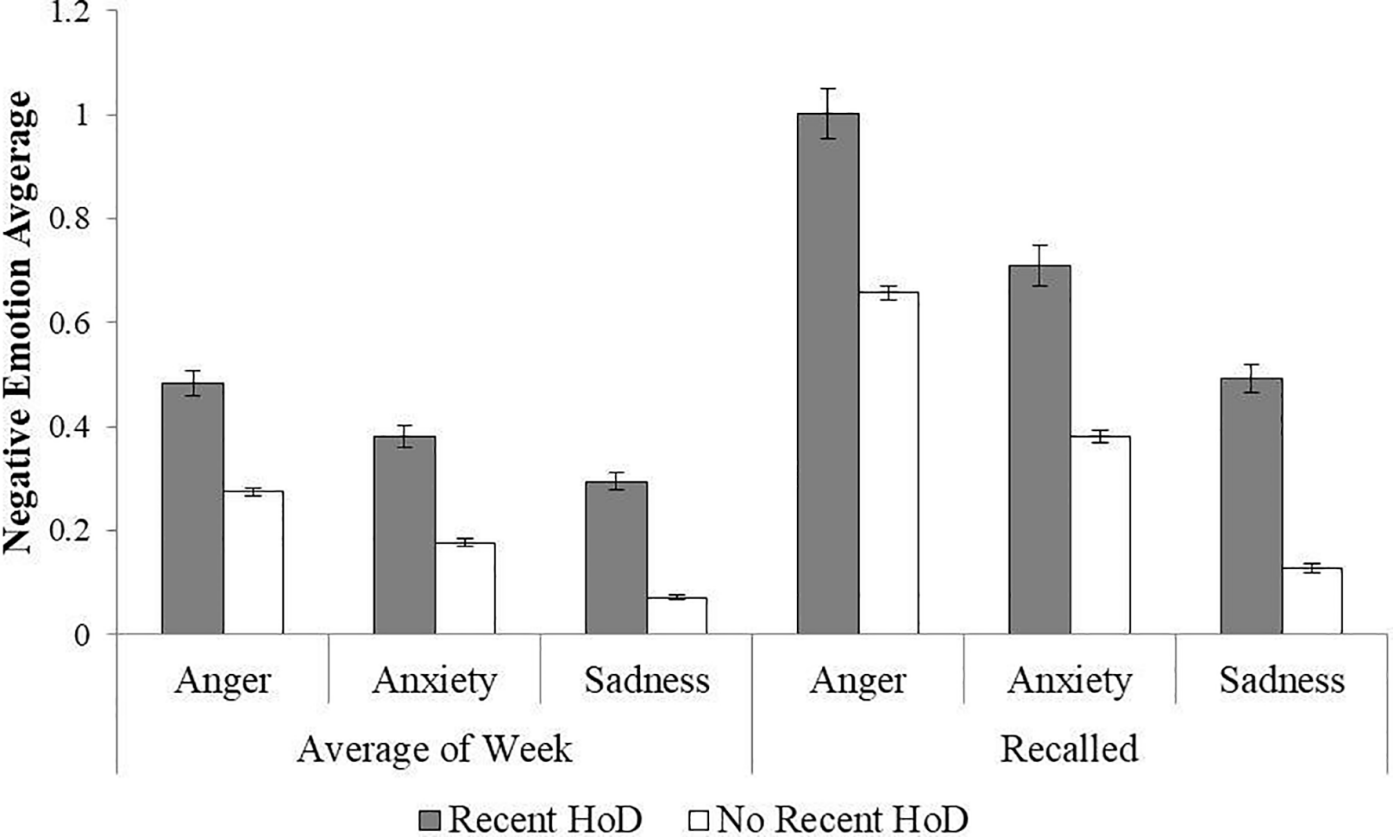
Negative clusters by recent history of depression. *Note*. Error bars represent standard error values. HoD = History of Depression. The model is adjusted for gender and evaluated at the following values: Age = 56.67, Education = 7.39.

A second RM GLM (see Table 3 for full results) included the within-person differences of negative emotion type (sadness vs. anxiety vs. anger) and reporting type (daily vs. weekly recollection) by between-subject factors of depression history (with vs. without) and age, gender, and education as covariates. Levels of sadness, anger, and anxiety were all significantly higher among those with a recent history of depression. As shown in Fig 2, anger was reported most by both groups (daily average: *M* = 0.38, *SE* = 0.01, *CI* [0.35, 0.40]), followed by anxiety (daily average: *M* = 0.28, *SE* = 0.01, *CI* [0.26, 0.30]), and sadness (daily average: *M* = 0.18, *SE* = 0.01, *CI* [0.17, 0.20]) as revealed by a main effect of cluster. Contrary to our hypothesis, the interaction between cluster and depression group was not significant indicating that this pattern did not differ significantly between the two groups.

**Table 3:** Effects of history of depression on experience and recall of sadness, anxiety, & anger.

		*F* (1, 1652)	*Partial η*^*2*^ [90% *CI*]
Within Subjects Main Effects	NACluster^a^	108.11***	.061 [.048, .075]
	Report Type	113.66***	.064 [.047, .084]
Between Subjects Main Effects	Age	63.53***	.037 .024, .053]
	Gender	10.47**	.006 [.002, .014]
	Education	6.03*	.004 [.000, .010]
	HoD	137.01***	.077 .057, .098]
2-way Interactions	NACluster *ReportType^b^	62.32***	.036 [.026, .048]
w/ NACluster	Age* NACluster^a^	122.17***	.069 [.055, .083]
	Gender* NACluster^a^	4.39*	.003 [.000, .006]
	Education*NACluster^a^	19.34***	.012 [.006, .018]
	HoD * NACluster^a^	0.43	.000 [.000, .002]
w/ Report Type	Age*ReportType	60.57***	.035 [.022, .051]
	Gender*ReportType	13.97***	.008 [.003, .017]
	Education*ReportType	13.78***	.008 [.003, .017]
	HoD *ReportType	38.46***	.023 [.012, .036]
3-way Interactions with Valence*ReportType	Age* NACluster*ReportType^b^	60.56***	.035 [.025, .047]
	Gender* NACluster*ReportType^b^	1.42	.001 [.000, .003]
	Education* NACluster*ReportType^b^	7.72**	.005 [.001, .009]
	HoD* NACluster*ReportType^b^	0.11	.000 [.000, .001]

*Note*. NACluster refers to negative affect cluster (anger, anxiety, or sadness). ReportType refers to daily emotion reports (averaged across the week) compared to recalled emotion (made at the end of the week). HoD = History of Depression. *CI* = Confidence interval. Due to violations to assumptions of sphericity, a Greenhouse-Geisser correction was used to adjust degrees of freedom for relevant tests.

^a^DF = (1.91, 3146.33).

^b^DF = (1.82, 2999.07).

***p < .001.

**p < .01.

*p < .05.

Recalled negative emotions were reported at higher levels than the daily reports, although this difference was greater for the group with a recent history of depression. Anger was overestimated more than either anxiety or sadness, as revealed by a significant interaction between emotion cluster and report type. There was no significant three-way interaction between history of depression, cluster, and report type. The results of this analysis revealed that experiences of anger are reported more frequently than sadness and anxiety regardless of depression status and that all three emotion clusters are elevated to a similar extent when comparing those with a recent history of depression to those without. In addition, all three emotion clusters tend to be overestimated in memory, but overestimation is greater for people with a history of depression and for memories of anger.

With respect to covariates, older age was related to experiencing less anger and anxiety during the week (anger: *r* = -.28, *p* <. .001, *CI* [-.34, -.24]; anxiety: *r* = -.08, *p* = .001, *CI* [-.13, -.03]) but was only moderately related to feeling less sadness, *r* = -.05, *p* = .063, *CI* [-.10, -.00]. Furthermore, with older age the difference between recalled and daily negative emotion was less pronounced for all three clusters (anger diff, *r* = -.27, *p* < .001, *CI* [-.31, -.23]; anxiety diff *r* = -.08, *p* = .001, *CI* [-.13, -.03]; sadness diff *r* = -.09, *p* < .001, *CI* [-.14, -.04]), but a significant three-way interaction indicated that older age was related to overestimating anger the least of the three clusters. Women tended to experience more of each negative emotion than men, *t*’s > 1.98, *p*’s < .048, *r*’s > .05, but experienced anger disproportionately more. Women also tended to overestimate each negative emotion at recall more than men, *t*’s > 2.68, *p*’s < .008, *r*’s > .07, which supports findings from the first RM GLM. Finally, higher levels of education were related to experiencing more anger, *r* = .10, *p* < .001, *CI* [.05, .15], and overestimating negative emotion in general more in memory, *r* = .09, *p* < .001, *CI* [.04, .14].

### Discussion

Memory for past emotions is susceptible to bias, and previous research has shown that depression may exacerbate this bias [9–12]. To our knowledge, only two studies have examined memory bias for daily emotions in relation to depression, and both examined aggregated levels of positive and negative emotion. No studies have examined memory bias for daily emotional experiences among people with a history of depression, who are at risk for recurrent depressive episodes [14]. The present study builds on previous work to examine experiences and memory biases for specific clusters of emotions (sadness, anxiety, and anger) as they occurred in daily life among people with and without a history of depression using a large, national sample of adults.

As expected, those who recently met the criteria for a history of depression reported lower levels of positive emotion, and higher levels of negative emotion, in their daily lives. All three clusters of negative emotions (anger, anxiety, and sadness) were elevated compared to the group without a depression history; strikingly, the difference between these two groups for the sadness-related emotions was not more pronounced than that of other emotions. In both groups, the frequency of anger was greater than that of sadness or anxiety. When examining recall bias, both groups significantly overestimated the frequency of negative, but not positive emotion. Those with a recent history of depression, however, overestimated their already elevated levels of negative emotion to a greater extent than did those without. Finally, both groups overestimated anger more than anxiety and sadness, showing that recollections of anger may be more susceptible to exaggeration.

### Elevation of sadness, anxiety, and anger

Sadness and anxiety are commonly associated with adult depression, and more recently the experience of anger has been acknowledged to accompany adult depression [23–26]. In the current study we found that people with a history of depression reported all three of these emotion clusters to a higher degree compared to those without a recent depression history, and this difference was of a similar magnitude for all three clusters. They did not, as predicted, report significantly higher levels of sadness-related emotions relative to the other emotions compared to the group without a recent depression history. This is particularly noteworthy given that anger is not currently included in the diagnosis of MDD and most empirical research on depression focuses on feelings of sadness and anxiety only. Our findings build upon a growing body of recent research showing that anger, along with sadness and anxiety, is a prominent experience for those with depression [37–38]. Our study extends prior research by providing daily reports of participants’ emotional experiences and demonstrates that emotions related to anger (including frustration, irritability, and feeling upset), along with sadness and anxiety, play a very important role in the daily experience of many with a history of depression.

### Recalling daily emotions

Participants in our sample tended to overestimate the frequency of daily emotions during recall, although significantly so only for negative emotions. When examining specific clusters of negative emotions, emotions related to the anger cluster were overestimated to a greater extent than sadness and anxiety; sadness and anxiety were overestimated to the same degree. Thus, people both experience more daily anger than sadness and anxiety and are more likely to overestimate these anger-related emotions in their recollections. These results suggest that feelings related to anger are more susceptible to biases in memory compared to other types of emotions. Anger is experienced when people appraise a situation as blocking a goal or perceive that they have been wronged [39]. Overestimating anger in memory (to a greater extent than other negative emotions), then, could serve as a stronger motivator to right the wrong.

Findings also indicate that people with a history of depression not only experienced more negative emotion on a daily basis, they also remembered their negative emotional experiences as occurring more frequently than what had actually occurred. This memory bias, which is consistent with prior research on cognitive biases associated with depression [7, 8, 11], can reinforce initial negative feelings, creating a double vulnerability for continuing symptoms of depression and increasing resistance to recovery.

Understanding the extent to which emotions are biased in memory is critical because memory about an emotional experience is often more predictive of future behavior than what was actually experienced [6]. For example, a study examining momentary emotions of college students during spring break found their intention to go on a similar trip in the future was more strongly predicted by the students’ memories of how they felt during their trip than their feelings during the vacation [40]. Another study found that the more students overestimated recollections of their pre-midterm exam anxiety, the more they planned to study for the final exam and the more anxious they felt just before taking the final exam, even after adjusting for the grade they received on the midterm [41]. These studies show that memories for previously experienced emotions can be a more powerful predictor of later judgments and behaviors than momentary reports of the same emotions.

In the context of the current study, recalling everyday emotional experiences as more negative than they were initially reported might make a person even less likely to engage in similar experiences in the future, despite the fact that the original experience might not have been very negative in the first place. For example, a person with a depressive history might recall a routine social interaction as a very negative experience. In actuality, they might have reported feeling a little down, self-conscious, or irritable in the moment- but not to the same extreme as they later recall feeling. These negatively biased memories may make the individual less likely to engage in similar social interactions the following week. Choosing *not* to interact in a similar social setting eliminates the possibility of experiencing further negative emotions, but also of experiencing any potential mood enhancing effects.

### Limitations

Of note, this study was limited by the fact that participants were administered the depression inventory an average of six months before the daily diary portion of the study. Given this time difference, some previously depressed individuals may not have been experiencing depressive symptoms during the diary week, and some previously non-depressed individuals may have developed new symptoms. Therefore, we cannot ascertain whether participants currently met the criteria for a depressive disorder at the time of the diary study. Despite this limitation, this measure does allow us to examine how people with a recent history of depression, whether they are currently experiencing an episode or not, might be at risk for a future episode. Our findings suggest that even a recent history of depression exerts significant effects on experienced and remembered emotions in daily life, and that these effects may be even larger in a sample of participants currently experiencing a depressive episode. The presence of a negative memory bias for daily emotion among people who have a history of depression, however, might help explain why depression is such a highly recurrent disorder.

Another limitation of our study is that the assessment of depression itself, similar to the assessment of depression in clinical settings, was retrospective in nature and thus also susceptible to memory bias. Future research could examine how reports of depression during the week compared to retrospectively might differ as a function of memory bias. Furthermore, our measure of daily experienced emotion was also technically retrospective in nature. Participants in this study were asked to think over the past day and report the emotions they felt during that time. It is possible that there may be some sort of retrospective bias that contributes to end-of-day emotion ratings as compared to other immediate measures, such as momentary sampling. However, previous research has found high correspondence between emotion reports collected using momentary sampling and the daily reconstruction method [18, 29]. Even if the retrospective nature of the present study’s daily reports of emotional experiences lead to bias, our results still demonstrate that the bias when recalling negative emotions at the end of a week is even greater. Despite this, future research could attempt to replicate these findings using a momentary design.

## Conclusions

Research presented here shows that sadness, anxiety, and anger are elevated to the same extent among people with a history of depression and that recollections of these emotions, particularly anger, are likely to be overestimated. Therefore people with a history of depression are not only more likely to *experience* more sadness, anxiety, and anger during daily life, but are also more susceptible to *remembering* these specific emotions as occurring even more frequently compared to people without a recent history of depression. These findings are important for forming an accurate picture of the daily lives of people with a history of depression and understanding what factors may contribute to vulnerability for recurrent depressive episodes.

## Supporting information

S1 Text Supplemental analyses of chronic depression history and memory bias. (DOCX)
