# Supplementary material for: Depression history and memory bias for specific daily emotions
Source: PLoS One. 2018 Sep 7;13(9):e0203574. doi: 10.1371/journal.pone.0203574 (PMC6128594; doi:10.1371/journal.pone.0203574)
Supplement: S1 Text — (DOCX) [file pone.0203574.s001.docx]

Seven hundred and eleven people participated in both MIDUS daily diary waves, had information about a history of depression at both waves, and completed the end-of-week emotion recall portion of the study on the final day of data collection. Of these participants, 38 met the criteria at wave 2 but not at wave 1 (recent history of depression) and 23 participants met the criteria both at wave 2 and ten years earlier (chronic history of depression). A repeated measures GLM was conducted to examine the mean duration of the negative emotion clusters and the discrepancy between report types among those with a chronic compared to recent history of depression.

This exploratory analysis yielded a main effect of depression, *F* (1, 55) = 7.52, *p* = .008, η^2^ = .12. The group with a chronic history of depression experienced even higher levels of anger, anxiety, and sadness than the group with only a recent history of depression. Regardless of depression group, anger was reported the most (Experienced: *M* = 0.54, *SE* = 0.06, *CI* [0.42, 0.66]; Recalled: *M* = 1.21, *SE* = 0.09, *CI* [1.03, 1.38]) followed by anxiety (Experienced: *M* = 0.40, *SE* = 0.07, [0.27, 0.53]; Recalled: *M* = 0.80, *SE* = 0.10, [0.59, 1.01]), and sadness (Experienced: *M* = 0.27, *SE* = 0.06, *CI* [0.16, 0.39]; Recalled: *M* = 0.52, *SE* = 0.08, CI[0.36, 0.69]), *F* (2, 110) = 6.46, *p* = .002, η^2^ = .105. Those with a chronic history of depression, however, overestimated in recalling their experience of all three emotion clusters to a greater extent than those with a recent history of depression, *F*(1, 55) = 7.20, *p* = .01, η^2^ = .116. These exploratory findings suggest that the experienced duration of negative emotions increases with the severity of depression history as does the tendency to overestimate the occurrence of these emotions.
